# The ubiquitin‐specific protease 5 mediated deubiquitination of LSH links metabolic regulation of ferroptosis to hepatocellular carcinoma progression

**DOI:** 10.1002/mco2.337

**Published:** 2023-07-22

**Authors:** Bokang Yan, Jiaxing Guo, Zuli Wang, Jieling Ning, Haiyan Wang, Long Shu, Kuan Hu, Ling Chen, Ying Shi, Lingqiang Zhang, Shuang Liu, Yongguang Tao, Desheng Xiao

**Affiliations:** ^1^ Department of Pathology Zhuzhou Hospital Affiliated to Xiangya School of Medicine Central South University Zhuzhou Hunan China; ^2^ Department of Pathology Xiangya Hospital Central South University Changsha Hunan China; ^3^ NHC Key Laboratory of Carcinogenesis (Central South University), Key Laboratory of Carcinogenesis and Cancer Invasion (Ministry of Education) Cancer Research Institute and School of Basic Medicine Central South University Changsha Hunan China; ^4^ Department of Hepatobiliary Surgery Xiangya Hospital Central South University Changsha Hunan China; ^5^ State Key Laboratory of Proteomics Beijing Proteome Research Center Beijing Institute of Radiation Medicine Collaborative Innovation Center for Cancer Medicine Beijing China; ^6^ Department of Oncology Institute of Medical Sciences National Clinical Research Center for Geriatric Disorders Xiangya Hospital Central South University Changsha Hunan China; ^7^ Department of Thoracic Surgery Hunan Key Laboratory of Early Diagnosis and Precision Therapy in Lung Cancer Second Xiangya Hospital Central South University Changsha Hunan China; ^8^ Department of Pathology School of Basic Medicine Central South University Changsha Hunan China

**Keywords:** epigenetics, ferroptosis, lymphoid‐specific helicase, posttranslational modifications, ubiquitin‐specific protease 5

## Abstract

Epigenetic regulators and posttranslational modifications of proteins play important roles in various kinds of cancer cell death, including ferroptosis, a non‐apoptotic form of cell death. However, the interplay of chromatin modifiers and deubiquitinase (DUB) in ferroptosis remains unclear. Here, we found that ubiquitin‐specific protease 5 (USP5) is regarded as a bona fide DUB of lymphoid‐specific helicase (LSH), a DNA methylation repressor, in hepatocellular carcinoma (HCC). Functional studies reveal that USP5 interacts with LSH and stabilizes LSH by a deubiquitylation activity‐dependent process. Furthermore, the USP5‐mediated deubiquitination of LSH facilitates the tumorigenesis of HCC by upregulating solute carrier family 7 member 11 (SLC7A11) to suppress ferroptosis of liver cancer cells. Moreover, the USP5 inhibitor degrasyn inhibits DUB activities of USP5 to LSH to suppress the progression of HCC. Additionally, USP5 and LSH are positively correlated and both are overexpressed and linked to poor prognosis in HCC patients. Together, our findings show that USP5 interacts with LSH directly and enhances LSH protein stability through deubiquitination, which, in turn, promotes the development of HCC by suppressing ferroptosis of liver cancer cells, suggesting that USP5 may be a potential therapeutic target for HCC.

## Graphical abstract

Ubiquitin‐specific protease 5 (USP5) could interact with chromatin remodeling protein lymphoid‐specific helicase (LSH) and stabilize LSH in a deubiquitylation activity‐dependent manner. USP5‐mediated deubiquitination of LSH inhibits ferroptosis to facilitate tumorigenesis of HCC through upregulating solute carrier family 7 member 11 (SLC7A11). Treatment of degrasyn targeting USP5 has an obvious effect on hepatocellular carcinoma (HCC). Our study that first reveals the effects of DUBs on epigenetic regulators is closely associated with the regulation of ferroptosis.

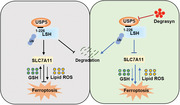

## 1 INTRODUCTION

Hepatocellular carcinoma (HCC) is one of the most common malignant tumor entities, ranking sixth in incidence and third in mortality worldwide.^1^ Due to the high occurrence of metastasis and recurrence of HCC, limited efficacy of radiotherapy and chemotherapy for the majority of HCC patients, and surgical treatment limited to early tumor stages, the underlying mechanism of HCC is urgently needed to be determined for the effective treatment of HCC.^2^, ^3^, ^4^, ^5^

Ferroptosis, a metabolism‐related and regulated cell death, predominantly brought on by intracellular iron catalytic activity and lipid peroxidation, is defined by intracellular lipid peroxide accumulation and redox imbalance.^6^, ^7^, ^8^, ^9^ Ferroptosis is involved in some degenerative diseases, various kidney diseases, liver diseases, brain injury, and stroke as a stress response.^7^ Meanwhile, numerous studies show that the great susceptibility of cancer cells to ferroptosis presents a rare opportunity for treating various kinds of cancers, especially in HCC.^10^, ^11^ Ferroptosis is one of the potential mechanisms of sorafenib in the treatment of HCC through the cystine/glutamate antiporter SLC7A11.^12^ Inhibition of regulators, such as retinoblastoma, nuclear factor erythroid 2‐related factor 2 (NRF2), and metallothionein‐1g (MT‐1G) that suppress the sorafenib‐induced ferroptosis, could improve the resistance of sorafenib, and the combination therapy of haloperidol with sorafenib also promotes the sorafenib‐induced ferroptosis.^13^ In addition, extracts and monomers from natural products, including Chinese herbs, provide new strategies for the induction of ferroptosis to inhibit HCC.^14^ Moreover, all these ferroptosis‐targeting drugs induce ferroptosis of HCC cells mainly through iron metabolism, system xc−/GSH/glutathione peroxidase 4 (GPX4), p53, lipid peroxidation, and p62‐Keap1‐Nrf2 pathway.^15^

Epigenetics, which determines gene transcription and has a significant impact on cell fate and developmental processes, mainly includes DNA methylation, histone modifications, noncoding RNAs, and chromatin remodeling.^16^, ^17^ Cancer cells frequently disrupt preexisting epigenetic systems to reprogram gene expression in a way that promotes the growth of cancer. Recently, many research have shown that a variety of epigenetic regulators, including lncRNA, deubiquitinase (DUB), and selenium, play crucial roles in ferroptosis by modulating metabolic genes and intermediaries, which results in a change in the level of lipid peroxidation.^15^

Lymphoid‐specific helicase (LSH), often referred to as helicase, lymphoid specific or proliferation‐associated SNF2‐like gene (PASG), an sucrose non‐fermenter (SNF2) chromatin remodeling enzyme,^18^ plays a vital role in preserving DNA methylation during the growth of plants and mammals.^19^, ^20^, ^21^, ^22^ In addition, LSH is crucial in repairing DNA‐damage to maintain the genome stability of somatic cells.^23^ Our previous research studies have shown that LSH also functions as a critical ferroptosis inhibitor in the tumorigenesis of lung cancer and leukemia.^24^, ^25^, ^26^ However, it is still unclear how LSH affects the ferroptosis of HCC. In addition, LSH is overexpressed in various cancers, such as melanoma, head‐and‐neck cancer, and prostate cancer, and its overexpression leads to poor prognosis in several cancers.^27^ Thus, LSH is probably involved in the cancer progression. However, the mechanism of LSH overexpression in cancers remains elusive, only a few studies have been conducted on it.^28^, ^29^ Moreover, the high LSH expression in tumor cells indicates the presence of LSH stabilizing factor.

Posttranslational modifications (PTMs) of proteins, which include ubiquitination, acetylation, SUMOylation and phosphorylation, play essential roles in regulating nearly all kinds of biological processes.^30^ Among them, ubiquitination, a common PTM reversible by reactions induced by DUBs, targets many oncogenes and tumor suppressor genes involved in the progression of cancers.^31^, ^32^ Ubiquitin‐specific protease 5 (USP5), a member of the USP family, the largest family of DUBs enzymes, also known as ubiquitin isopeptidase T, is distinctive in that it can specifically identify and remove ubiquitin from the proximal end of the unanchored polyubiquitin chains.^33^, ^34^ Multiple cellular activities, including the repair of DNA double‐strand breaks, inflammatory responses, and stress responses, are regulated by USP5.^35^, ^36^, ^37^ In this study, we reported that USP5 is an inhibitor of ferroptosis of HCC cells that promotes the development of HCC through stabilizing LSH protein. We revealed a previously unappreciated regulatory mechanism of LSH and the significant inhibitory effect of degrasyn on the tumorigenesis of HCC. Our study may provide a novel antitumor therapy of HCC by targeting USP5.

## 2 RESULTS

### 2.1 USP5 interacts with LSH

**FIGURE 1:**
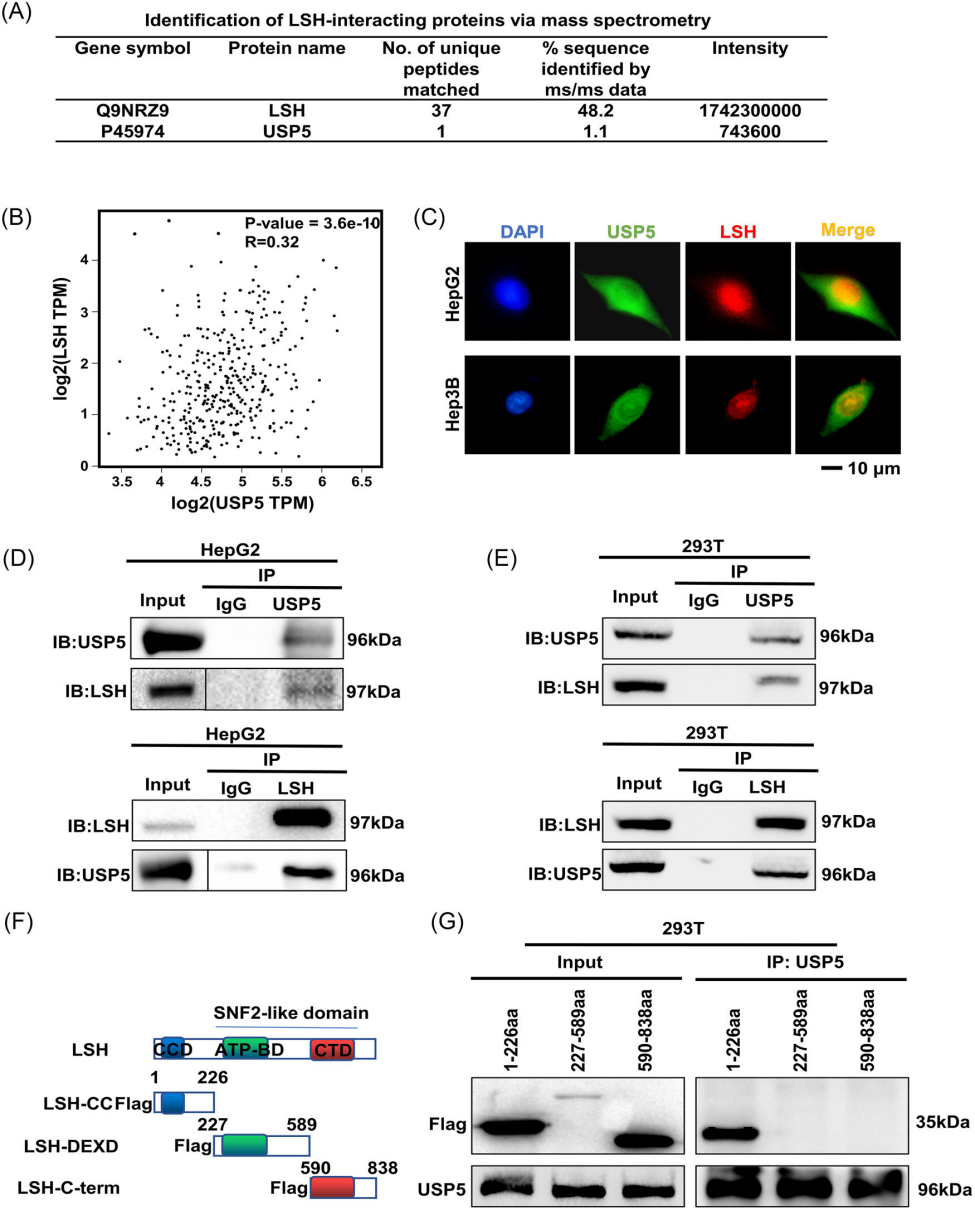
Ubiquitin‐specific protease 5 (USP5) interacts with lymphoid‐specific helicase (LSH). (A) The particular interaction information between USP5 and LSH was shown by the mass spectrometry analysis of LSH‐associated proteins in 293T cells. (B) Correlation analysis of mRNA level between USP5 and LSH in the Cancer Genome Atlas (TCGA), hepatocellular carcinoma (HCC) samples from Expression Profiling Interactive Analysis (GEPIA). (C) Immunofluorescence assay of the co‐localization of USP5 and LSH in HepG2 and Hep3B cells. (D and E) Analysis of the endogenous or exogenous interaction between USP5 and LSH using coimmunoprecipitation (Co‐IP) and immunoblotting (IB). HepG2 (D) and 293T (E) were immunoprecipitated followed by IB with anti‐USP5 or anti‐LSH antibody. (F and G) Structure of LSH and FLAG‐LSH fragments, including LSH‐CC (coiled coil), LSH‐DEXD (DEAH box domain), and LSH‐C‐term (helicase C‐terminal) (F). For interaction between LSH truncation mutants and full‐length USP5, IP analysis was followed by an IB assay (G).

To completely clarify its potential regulatory mechanism, LSH‐interacting proteins were identified using mass spectrometry in 293T cells stably expressing LSH.^38^ LSH was found to be associated with numerous proteins, including USP5 (Figure 1A). Then the correlation analysis between USP5 and LSH in the Cancer Genome Atlas (TCGA) HCC samples from Expression Profiling Interactive Analysis (GEPIA) revealed that USP5 and LSH were positively associated (Figure 1B). USP5 and LSH colocalized mainly in the nucleus of HepG2 and Hep3B cells according to the immunofluorescence assay (Figure 1C). To further demonstrate this interaction between USP5 and LSH, coimmunoprecipitation experiments were performed, and the results showed that USP5 could coimmunoprecipitate with LSH endogenously in HepG2 cells and exogenously in 293T cells (Figure 1D,E). Moreover, deletion analyses were generated to map the domains of USP5 and LSH responsible for the interaction, and we discovered that the interaction between USP5 and LSH depended on the N‐terminus of LSH (Figure 1F,G). In conclusion, these data show that USP5 can directly interact with LSH.

### 2.2 USP5 stabilizes the LSH protein through deubiquitination

**FIGURE 2:**
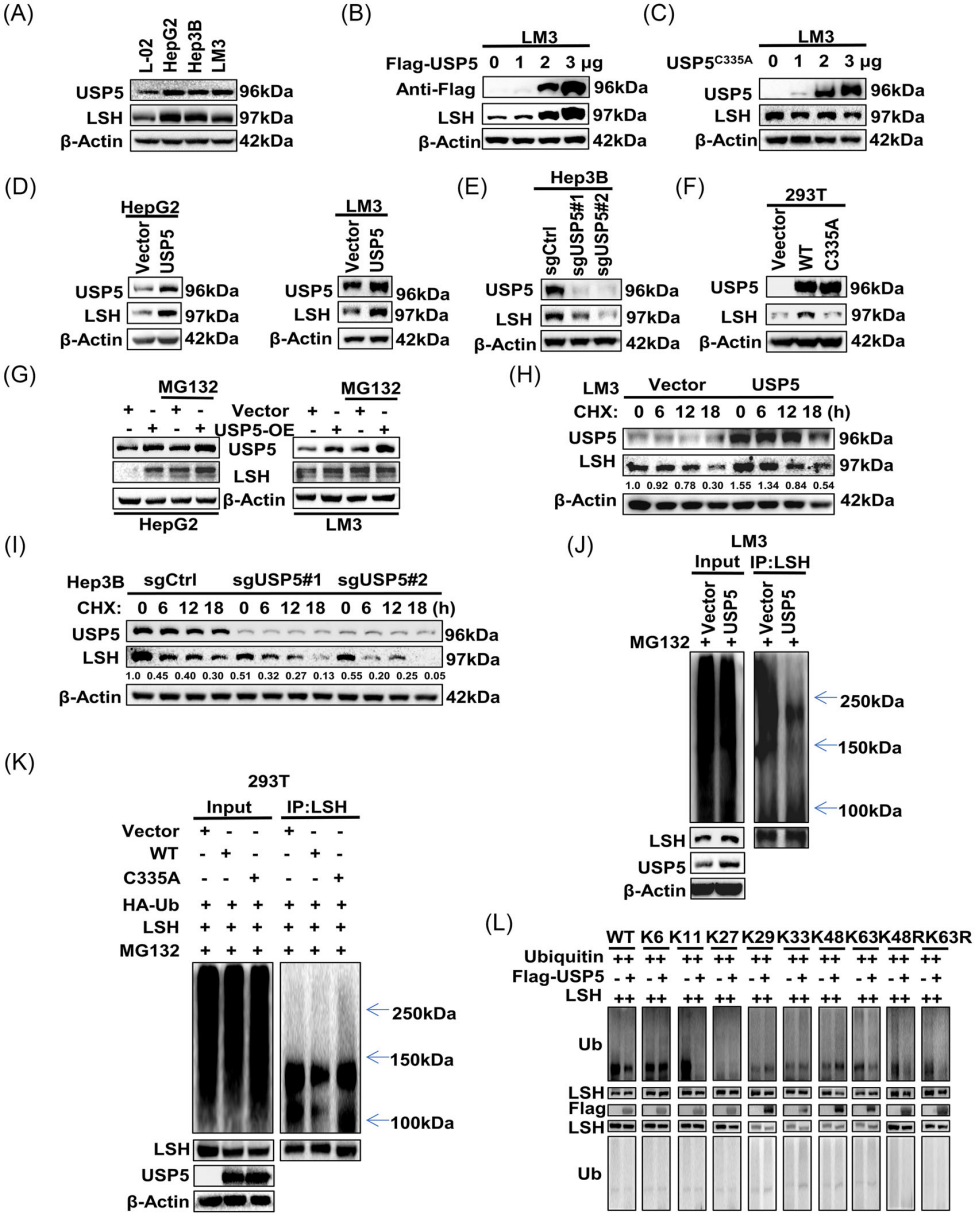
Ubiquitin‐specific protease 5 (USP5) stabilizes the lymphoid‐specific helicase (LSH) protein through deubiquitination. (A) Western blot for detecting the protein level of USP5 and LSH in hepatocellular carcinoma (HCC) and normal liver cell lines. (B and C) Western blot was performed to detect the expression of LSH after increasing amounts of USP5 WT (B) or C335A (C) plasmids were transfected into LM3 cells. (D) Western blot for detecting the expression level of LSH in HepG2 and LM3 cells after the overexpression of USP5. (E) Western blot for detecting the expression level of LSH in Hep3B cells after knockout of USP5. (F) After transfecting 293T cells with the USP5‐C335A plasmid, the LSH expression level was assessed using western blot. (G) Stable USP5‐overexpressing HepG2 and LM3 cells were treated with or without MG132 (20 μM, 12 h), and the protein level of LSH was detected using western blot. (H and I) LM3 (H) cells stably overexpressing USP5 or Hep3B (I) cells knockout of USP5 were treated with cycloheximide (CHX, 10 mg/mL) for the indicated time followed by WB. (J) Before collection, MG132 (20 μM, 12 h) was applied to LM3 cells stably overexpressing USP5. Anti‐LSH and anti‐Ub antibodies were used to immunoprecipitate and immunoblot LSH. (K) 293T cells co‐transfected with LSH, HA‐ubiquitin, and USP5 (WT or C335A) were treated with MG132 (30 μM, 8 h), followed by western blot to detect the ubiquitinated LSH protein levels. (L) After treating with MG132 (20 μM, 12 h), 293T cells that were co‐transfected with LSH, USP5 and WT, K6, K11, K27, K29, K33, K48, K63, K48R, or K63R were collected to subjected to ubiquitination assay, and anti‐Ub was used to detect the ubiquitination level of LSH.

To further investigate the relationship between USP5 and LSH, we first examined the levels of protein expression of USP5 and LSH in normal liver L‐02 cells and several liver cancer cells, and the results showed that USP5 was significantly expressed and positively correlated with LSH in HCC cells (Figure 2A). Many studies had proved that C335 is the key catalytic residue of USP5.^39^, ^40^, ^41^ In our study, USP5‐WT overexpression elevated LSH expression in LM3 cells, but not USP5‐C335A (Figure 2B,C). In addition, a stable overexpression of USP5 in HepG2 and LM3 increased the protein expression of LSH (Figure 2D), knockout of USP5 in Hep3B induced the reduction of LSH protein level (Figure 2E), and the level of LSH did not change after transfecting USP5‐C335A into 293T (Figure 2F). Moreover, the mRNA level of LSH did not change (Figure S1A), and the change of the LSH protein expression due to USP5 overexpression or knockout could be rescued by proteasome inhibitor Z‐Leu‐Leu‐Leu‐al (MG132)^42^ (Figure 2G, Figure S1B), but not restored by lysosomal inhibitor chloroquine (CQ)^43^ (Figure S1C). Meanwhile, cycloheximide (CHX)^44^ chase assays showed that the half‐life of LSH was prolonged by the overexpression of USP5 and decreased in USP5‐knockout cells (Figure 2H,I, Figure S1D). Thus, we confirmed that USP5 inhibited LSH degradation to stabilize LSH protein through proteasome pathway. As USP5 is a DUB, we continued to test whether USP5‐regulated LSH protein stability via deubiquitination. The DUB assays demonstrated that the ubiquitin level of LSH was decreased in USP5‐overexpressed cells, but did not happen in USP5‐C335A‐overexpressed 293T cells, and knockout of USP5 increased the ubiquitination of endogenous LSH (Figure 2J,K, Figure S1E–G). Furthermore, the experiments to detect which types of ubiquitin of LSH were deubiquitylated by USP5 showed that USP5 significantly cleaved the K11‐linked ubiquitin chain and slightly removed the K63‐linked ubiquitin chain from LSH (Figure 2L). Taken together, we found that USP5 stabilized the LSH protein through DUB LSH.

### 2.3 Overexpression of USP5 inhibits ferroptosis

**FIGURE 3:**
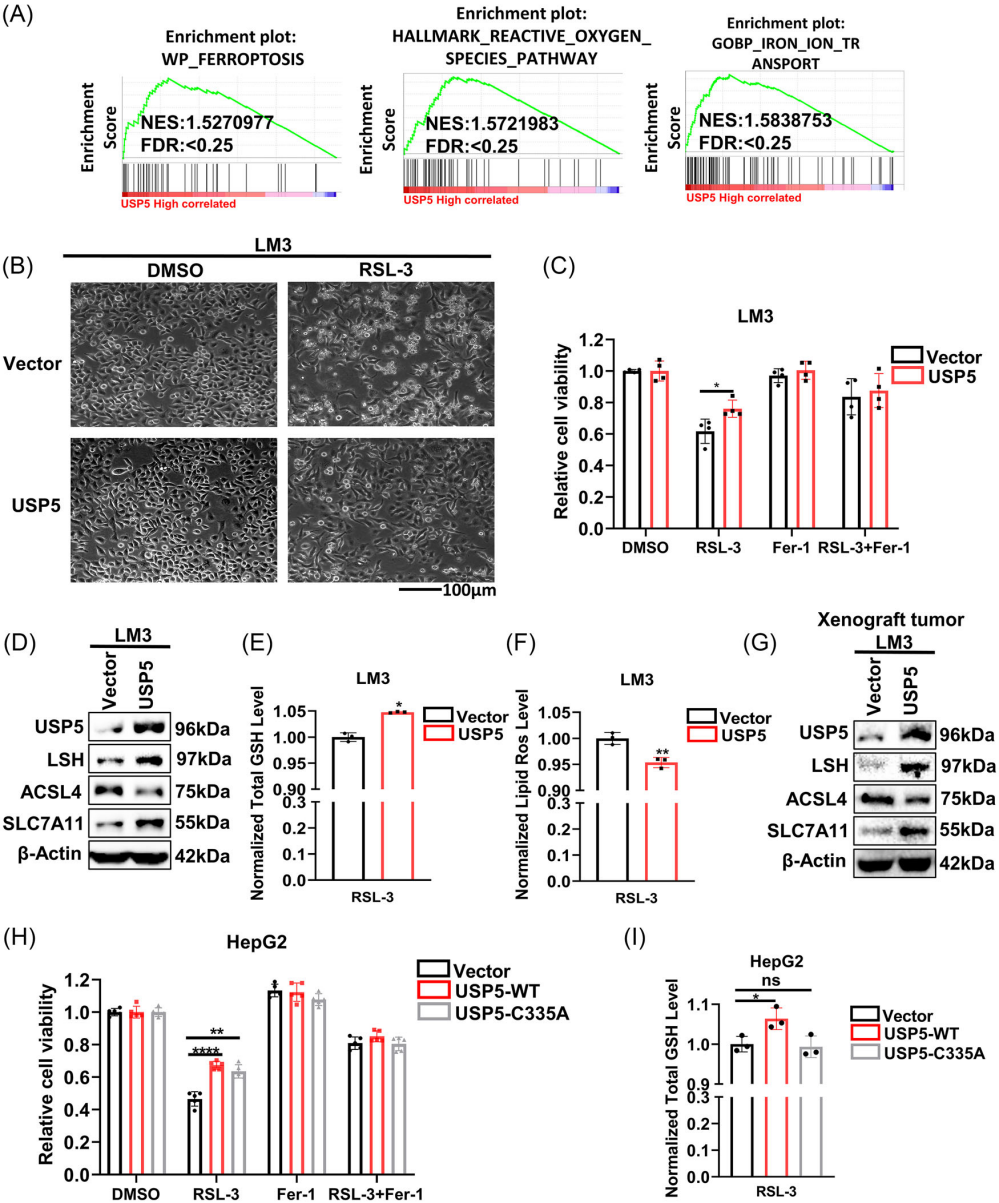
Overexpression of ubiquitin‐specific protease 5 (USP5) inhibits ferroptosis. (A) The effect of USP5 on ferroptosis based on gene set enrichment analysis (GSEA) (“WikiPathways [WP] Ferroptosis,” “Hallmark reactive oxygen species pathway,” and “Gene Ontology Biological Process [GOBP] iron ion transport”). (B) Representative phase‐contrast images of LM3 cells stably overexpressing USP5 treated with RSL‐3 (10 μM, 24 h). (C) Analysis of the responses of LM3 cells overexpressing USP5 to ferrostatin (Fer‐1) (10 μM, 24 h) and RSL‐3 (10 μM, 24 h) was done using cell counting kit‐8 (CCK8) assay. (D) The expression level of lymphoid‐specific helicase (LSH) and ferroptosis‐related proteins are detected using western blot in LM3 cells stably overexpressing USP5. (E and F) The levels of total GSH (E) and lipid ROS (F) in LM3 cells stably overexpressing USP5 were analyzed. (G) Western blot was used to test the ferroptosis‐related proteins in xenograft tumors from LM3 cells overexpressing USP5. (H) The response of HepG2 cells transfected with USP5‐WT or USP5‐C335A to ferrostatin (10 μM, 24 h) and RSL‐3 (10 μM, 24 h) was analyzed by CCK8 assays. (I) The levels of total glutathione (GSH) in HepG2 cells transfected with USP5‐WT or USP5‐C335A were analyzed. Ns, nonsignificant (*p* > 0.05), *p < 0.05, **p < 0.01, and ****p < 0.0001.

In our previous studies, we demonstrated that LSH inhibits ferroptosis in lung cancer and leukemia by reducing the intracellular concentrations of iron and lipid ROS.^24^, ^26^ However, the function of LSH in ferroptosis of HCC remains unclear. To address whether USP5 could regulate ferroptosis by stabilizing LSH in HCC, we first used gene set enrichment analysis to explore the effects of USP5 on ferroptosis in HCC, and the results revealed that “WikiPathways Ferroptosis,” “reactive oxygen species pathway,” and “iron ion transport” showed enrichment differences between high and low USP5 expression group (Figure 3A). Then we treated LM3 cells and HepG2 cells with RSL‐3,^45^ and the results of morphological observations showed the percentage of dead cells induced by RSL‐3 decreased after USP5 overexpression (Figure 3B, Figure S2A). Moreover, the cell counting kit‐8 (CCK8) analysis indicated that USP5 overexpression inhibited the RSL3‐induced ferroptosis of LM3 cells and HepG2 cells (Figure 3C, Figure S2B). Western blot to detect the ferroptosis‐associated markers revealed that acyl‐coenzyme A synthetase long‐chain family member 4 (ACSL4), one of the ferroptosis promoters, was decreased by USP5 overexpression. Meanwhile, solute carrier family 7 member 11 (SLC7A11), the cystine/glutamate antiporter, known as a key suppressor of ferroptosis, was increased by the overexpression of USP5 (Figure 3D, Figure S2C). Both of the intracellular levels of lipid ROS and GSH were surrogate markers for ferroptosis. We found that after treating cells with RSL‐3, the overexpression of USP5 increased GSH levels and decreased the lipid ROS levels in LM3 cells and HepG2 cells (Figure 3E,F, Figure S2D,E). And the detection of ferroptosis‐associated markers in xenograft tumor samples with USP5 overexpression also showed that ACSL4 decreased, and SLC7A11 increased (Figure 3G). However, after treating HepG2 cells transfected with USP5‐C335A with RSL‐3, the ferroptosis level of HepG2 cells did not show an obvious change indicated by CCK8 and the detection of GSH and lipid ROS (Figure 3H,I, Figure S2E).

### 2.4 Knockout of USP5 promotes ferroptosis

**FIGURE 4:**
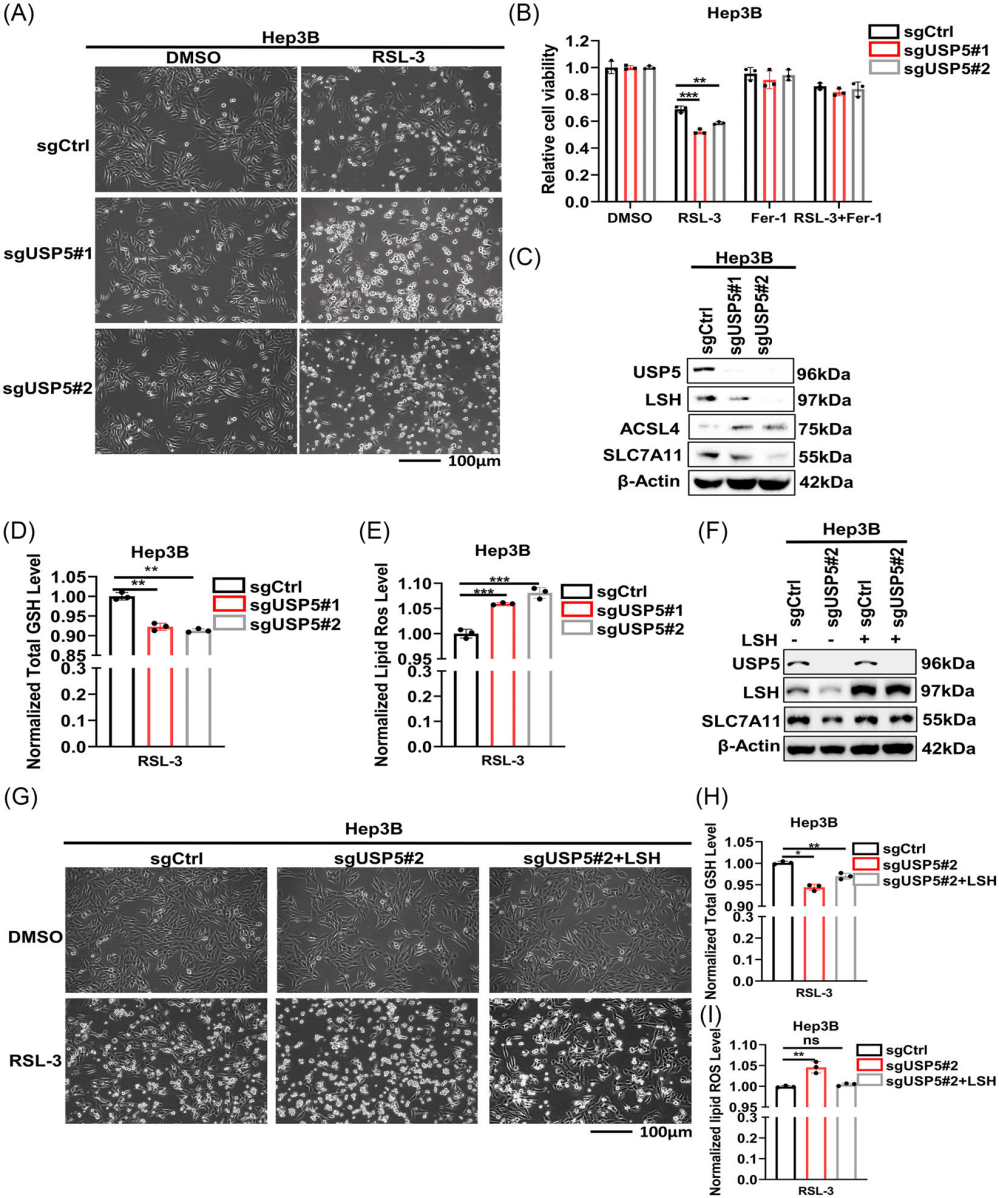
Knockout of ubiquitin‐specific protease 5 (USP5) promotes ferroptosis. (A) Representative phase‐contrast images of Hep3B cells knockout of USP5 treated with RSL‐3 (10 μM, 24 h). (B) Analysis of the responses of Hep3B cells knockout of USP5 to Fer‐1 (10 μM, 24 h) and RSL‐3 (10 μM, 24 h) was done using cell counting kit‐8 (CCK8) assays. (C) The expression level of lymphoid‐specific helicase (LSH) and ferroptosis‐related proteins were detected using western blot in Hep3B cells knockout of USP5. (D and E) The levels of total GSH (D) and lipid ROS (E) in Hep3B cells knockout of USP5 were analyzed. (F) Western blot was used to detect the expression level of LSH and SLC7A11 in Hep3B cells knockout of USP5 with LSH overexpression. (G) Representative phase‐contrast images of Hep3B cells knockout of USP5 with LSH overexpression treated with RSL‐3 (10 μM, 24 h). (H and I) The levels of total GSH (H) and lipid ROS (I) in Hep3B cells knockout of USP5 with LSH overexpression were analyzed. Ns, nonsignificant (*p* > 0.05), *p < 0.05, **p < 0.01, and ***p < 0.001.

After treating Hep3B cells with RSL‐3, we found that the percentage of dead cells induced by RSL‐3 increased after the knockout of USP5 by morphological observation (Figure 4A). Meanwhile, the knockout of USP5 promoted the ferroptosis level of Hep3B cells indicated by the CCK8 analysis (Figure 4B). And the results of western blot showed that ACSL4 increased, and SLC7A11 decreased after the knockout of USP5 (Figure 4C). Moreover, the detection of GSH and lipid ROS of Hep3B cells treated with RSL‐3 delivered that knockout of USP5 could decrease the GSH level and increase the lipid ROS level of Hep3B cells (Figure 4D,E). Furthermore, we observed that the promotions of ferroptosis induced by knockout of USP5 were rescued by overexpressing LSH in USP5‐knockout Hep3B cells (Figure 4F–I). Taking together, our results demonstrated that USP5 inhibited ferroptosis by stabilizing LSH protein to a certain extent in HCC.

### 2.5 USP5 promotes tumor growth dependent on stabilizing LSH

**FIGURE 5:**
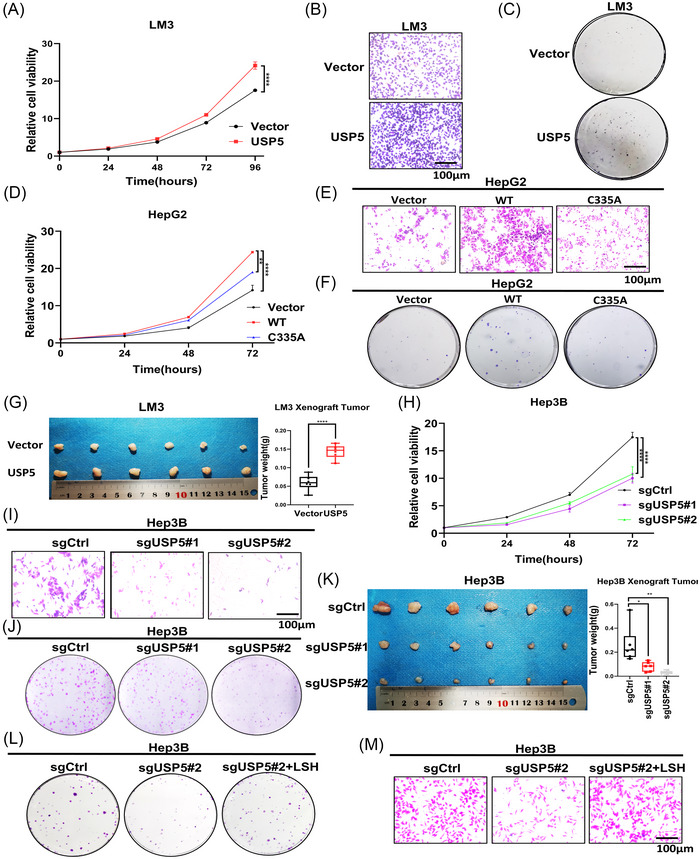
Ubiquitin‐specific protease 5 (USP5) promotes tumor growth dependent on stabilizing lymphoid‐specific helicase (LSH). (A–C) The cell counting kit‐8 (CCK8) assay (A), transwell assay (B), and colony formation assay (C) of LM3 cells stably overexpressing USP5. Scale bars, 100 μm. (D–F) The CCK8 assay (D), transwell assay (E), and colony formation assay (F) of HepG2 cells transfected with USP5‐C335A. Scale bars, 100 μm. (G) LM3 cells stably overexpressing USP5 were transplanted on nude miced (*n* = 6 mice per group). (H–J) The CCK8 assay (H), transwell assay (I), and colony formation assay (J) of Hep3B cells knockout of USP5. Scale bars, 100 μm. (K) Hep3B cells knockout of USP5 were transplanted on nude mice (*n* = 6 mice per group). (L and M) The colony formation assay (L) and transwell assay (M) of Hep3B cells knockout of USP5 with LSH overexpression. Scale bars, 100 μm. *p < 0.05, **p < 0.01, and ****p < 0.0001.

To investigate the effect of USP5 in HCC tumor growth and migration, USP5‐overexpressed HepG2 and LM3 cells were established to perform the gain‐of‐function studies. We found that cell proliferation, cell migration, and colony formation ability of HepG2 (Figure S3A–C) and LM3 cells (Figure 5A–C, Figure S4A,B) increased upon the overexpression of USP5, indicating that USP5 promotes HCC growth and migration. However, the HepG2 cells transfected with USP5‐C335A did not show the increased ability of proliferation and migration (Figure 5D–F, Figure S4C,D). To further confirm the oncogenic functions of USP5 on HCC in vivo, USP5‐overexpressed LM3 cells were subcutaneously transplanted into nude mice. In this experiment, tumor size and weight were obviously increased in mice with USP5 overexpression (Figure 5G, Figure S4E). These findings suggested that the growth of HCC is promoted by USP5 overexpression. Additionally, the loss‐of‐function study demonstrated that USP5 deletion markedly reduced cell proliferation, migration, and colony formation in Hep3B cells (Figure 5H–J, Figure S4F,G). And tumor growth was suppressed in nude mice receiving USP5 knockout transplants (Figure 5K, Figure S4H). In order to further detect whether the oncogenic functions of USP5 on HCC worked through stabilizing LSH, we first transiently overexpressed LSH in Hep3B cells stably knockout of USP5. The results showed that LSH overexpression efficiently rescued the cell growth and migration arrests induced by the depletion of USP5 (Figure 5L,M, Figure S4I,J). Then we transiently knockdown LSH in Hep3B cells overexpressing of USP5 to further prove the LSH‐dependent oncogenic role of USP5, and we found that the knockdown of LSH expression could decrease the oncogenic function of USP5 (Figure S4K–N). In summary, we demonstrated that USP5 promoted liver cancer cells proliferation dependent on stabilizing LSH.

### 2.6 Degrasyn promotes ferroptosis to inhibit tumor progression via targeting of USP5

**FIGURE 6:**
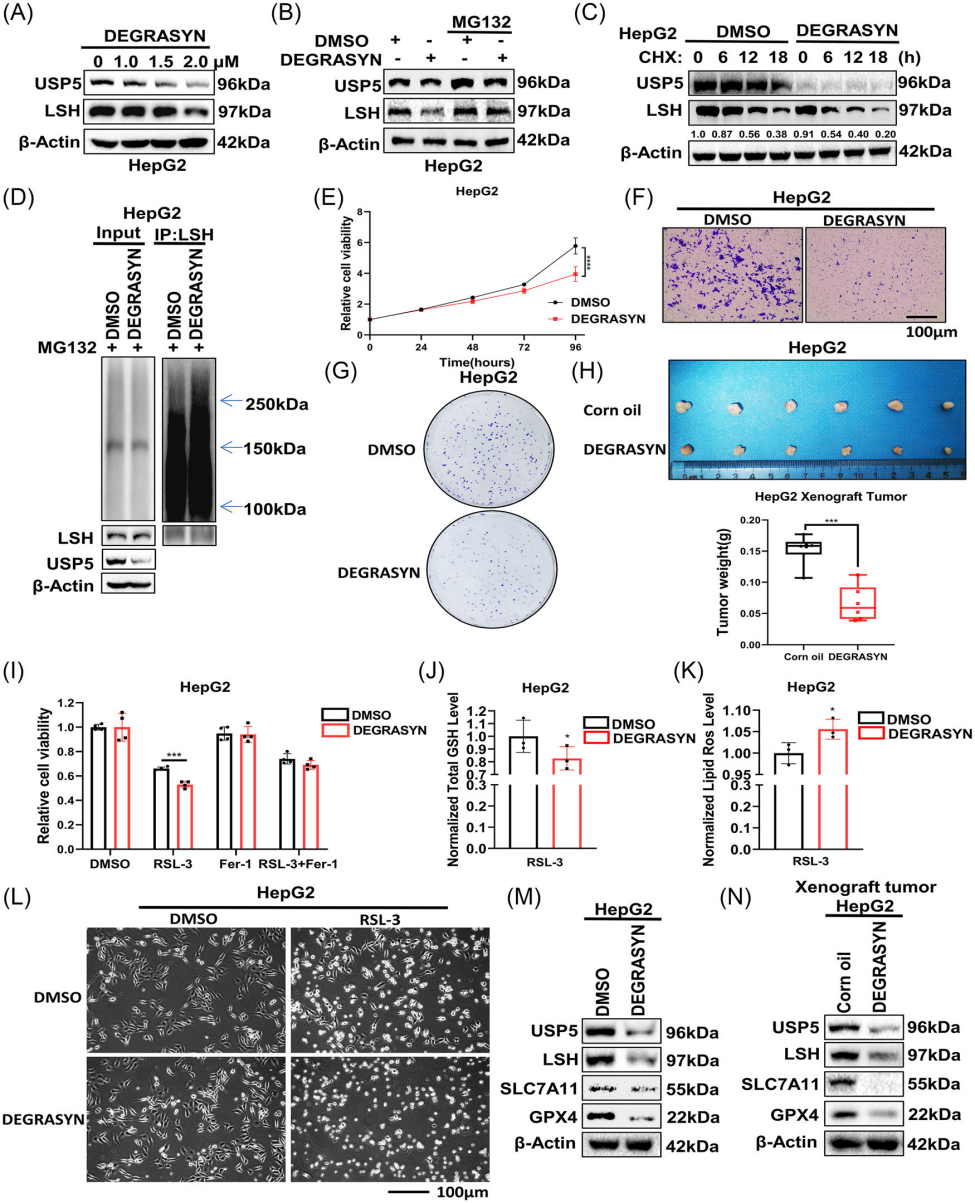
Degrasyn promotes ferroptosis to inhibit tumor progression via targeting of ubiquitin‐specific protease 5 (USP5). (A) Western blot was used to detect the lymphoid‐specific helicase (LSH) expression in HepG2 cells treated with degrasyn at different concentrations. (B) HepG2 cells treated with degrasyn were treated with or without MG132 (20 μM, 12 h), then western blot was used to test the protein level of LSH. (C) HepG2 cells treated with degrasyn were treated with cycloheximide (CHX, 10 mg/mL) for the indicated time followed by WB. (D) Before being collected, degrasyn‐treated HepG2 cells were given MG132 (20 μM, 12 h) treatment. Anti‐LSH was used to immunoprecipitate LSH, and anti‐Ub was used for immunoblotting. (E–G) The cell counting kit‐8 (CCK8) assay (E), transwell assay (F), and colony formation assay (G) of HepG2 cells treated with DMSO or degrasyn. Scale bars, 100 μm. (H) The parental HepG2 cells were transplanted on nude mice treated with corn oil or degrasyn intraperitoneally (25 mg/kg, 3 times/week) (*n* = 6 mice per group). (I) CCK8 assays were used to analyze the responses of HepG2 cells treated with degrasyn to Fer‐1 (10 μM, 24 h) and RSL‐3 (10 μM, 24 h). (J and K) The levels of total GSH (J) and lipid ROS (K) were analyzed in HepG2 cells treated with degrasyn. (L) Representative phase‐contrast images of degrasyn‐treated HepG2 cells treated with RSL‐3 (10 μM, 24 h). (M and N) Western blot was used to detect LSH and ferroptosis‐related proteins in LM3 cells degrasyn‐treated HepG2 cells (M) and xenograft tumor samples (N). *p < 0.05, ***p < 0.001, and ****p < 0.0001.

We used degrasyn, a specific DUB inhibitor, to further confirm our findings.^46^ First, we treated HepG2 cells with degrasyn at increasing concentrations,^47^ and we found that both USP5 and LSH decreased with increasing inhibitor concentration (Figure 6A). In addition, the reduction of LSH caused by degrasyn could be rescued by MG132 (Figure 6B), but not CQ (Figure S5A), and co‐treatment of CHX and degrasyn decreased the level of LSH more quickly (Figure 6C). Deubiquitination assay also indicated that ubiquitin of LSH increased in degrasyn‐treated HepG2 cells (Figure 6D). These results showed that degrasyn reduced USP5 to promote the ubiquitination of LSH. We also found that USP5 inhibitor degrasyn suppressed cell proliferation, cell migration, and colony formation ability of HepG2 cells (Figure 6E–G, Figure S5B,C). Then, in order to prove that the promotion effect of USP5 on the development of liver cancer was a common phenomenon in HCC and the potential value of targeting USP5 in the treatment of HCC, we used the xenograft nude mice model composed of another live cell line HepG2 cells to further reveal that the treatment of degrasyn targeting USP5 resulted in a decrease of the HCC tumor growth (Figure 6H, Figure S5D). Furthermore, we demonstrated that in HepG2 cells treated with RSL‐3, degrasyn could target USP5 to suppress cell growth, increase lipid ROS, and decrease GSH level (Figure 6I–K). Moreover, the dead cells induced by RSL‐3 increased after the treatment of degrasyn through morphological observations (Figure 6L). Meanwhile, western blot to detect ferroptosis‐associated markers of degrasyn‐treated HepG2 cells (Figure 6M) and xenograft tumor samples (Figure 6N) showed that key markers of ferroptosis, SLC7A11, and GPX4 decreased obviously. Conclusively, all these results revealed that degrasyn could target USP5 to promote the ferroptosis in HCC.

### 2.7 USP5 is upregulated and associated with poor survival in HCC

**FIGURE 7:**
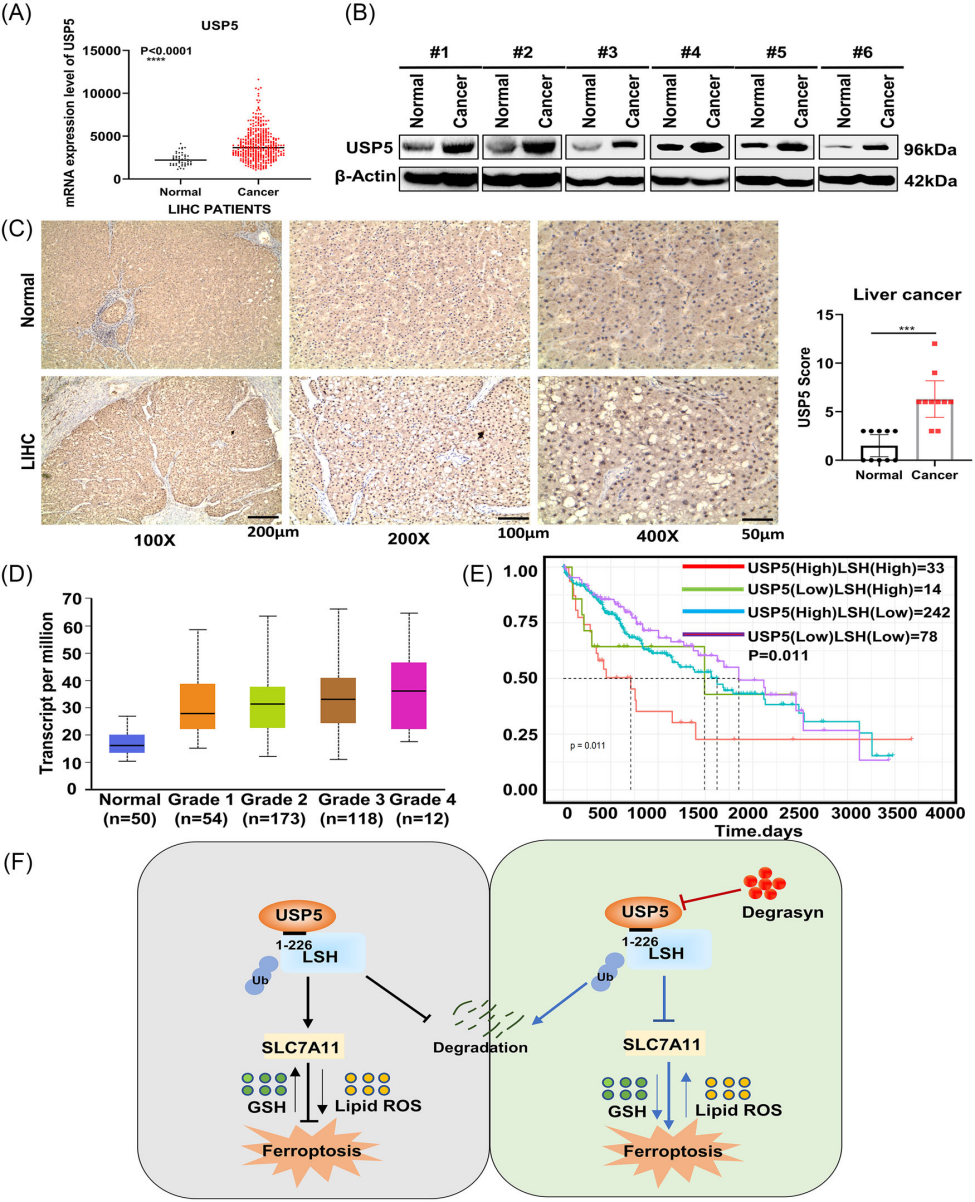
Ubiquitin‐specific protease 5 (USP5) is upregulated and associated with poor survival in hepatocellular carcinoma (HCC). (A) USP5 mRNA expression levels in HCC samples (*n* = 374) and normal liver tissues (*n* = 50) were analyzed using the Cancer Genome Atlas (TCGA). (B) In six pairs of HCC tissues and normal liver tissues, USP5 protein expression levels were detected using western blot. (C) Immunohistochemistry (IHC) test of USP5 protein expression in 10 pairs of clinical samples (magnification, ×100, scale bar = 200 μm; magnification, ×200, scale bar = 100 μm; magnification, ×400, scale bar = 50 μm). (D) The expression of USP5 in TCGA liver HCC (LIHC) based on tumor grade (*n* = 374). (E) According to USP5 and lymphoid‐specific helicase (LSH) expression, the TCGA LIHC samples’ overall survival rate is represented by a Kaplan–Meier curve (*n* = 367). (F) A working model of deubiquitination of LSH by USP5 in inhibiting ferroptosis drawn by PowerPoint. USP5 inhibits ferroptosis through the stabilization of LSH in a deubiquitinase (DUB)‐dependent manner. Moreover, the increased LSH regulates the expression of ferroptosis‐related proteins. And the ability of USP5 to stabilize LSH can be inhibited by degrasyn. ***p < 0.001 and ****p < 0.0001.

Last, we analyzed the mRNA expression of USPs family to find that among these USPs, USP5 obviously increased in HCC samples compared with normal liver tissue (Figure 7A, Figure S6A). Moreover, western blot for detecting the expression level of USP5 in six pairs of HCC tissues and para‐tumor tissues further demonstrated that USP5 was significantly higher in tumor tissues (Figure 7B). Moreover, immunohistochemistry (IHC) staining for USP5 and LSH showed that both of them were elevated in HCC, and the positive staining of USP5 was located in the nucleus and cytosol (Figure 7C, Figure S6B). In addition, USP5 was positively correlated with tumor grade of TCGA HCC samples (Figure 7D). We performed survival analysis in HCC patients, and the results showed that a poor prognosis was linked to the high expression of USP5 and LSH (Figure S6C,D), and patients with high expression of both USP5 and LSH showed the worst prognosis (Figure 7E).

## 3 DISCUSSION

The levels of chromatin remodeling enzyme LSH are elevated and associated with poor clinical outcomes in various cancers, and substantial evidence suggests that LSH promotes the malignant progression of tumor cells.^38^, ^48^ A deeper understanding of the upstream regulatory mechanism of LSH is important for the discovery of new target for the treatment of cancer. DUBs protect proteins from degradation by removing ubiquitin from substrates.^49^ Here, we have identified USP5 as a bona fide regulator of LSH. First, USP5 interacts with LSH directly. Second, USP5 enhances LSH protein stabilization through deubiquitination, and this ability could be inhibited by degrasyn, an USP5 inhibitor. Finally, USP5 could inhibit ferroptosis through DUB LSH to promote tumor progression.

Deubiquitination is relevant for the occurrence and development of tumorigenesis.^50^, ^51^ Many researches have proved that DUBs involve in the regulation of some cancer‐related pathways, such as Akt (protein kinase B), Wnt/β‐catenin signaling, transforming growth factor‐β, and NF‐κB (nuclear factor kappa‐light‐chain‐enhancer of activated B cells).^52^, ^53^, ^54^, ^55^ Meanwhile, multiple studies suggest that the effects of DUBs on ferroptosis‐related proteins could be a new epigenetic therapy for cancer treatment. To date, all DUBs regulating ferroptosis through the inhibition of system Xc‐/GPX, such as OTU DUB, ubiquitin aldehyde binding 1 (OTUB1), could directly interact with and stabilize SLC7A11 to inhibit ferroptosis, and BRCA1 associated protein 1 can decrease the amount of H2Aub occupying the SLC7A11 promoter to inhibit SLC7A11 expression, thereby inhibiting cystine uptake, resulting in increased levels of lipid peroxidation.^8^, ^56^ Moreover, it has been found that the inhibition of DUBs could be a potential anticancer therapy through the induction of GPX4 proteasomal degradation.^57^ However, the number of studies on the discovery of DUBs targeting ferroptosis‐related proteins is still limited.

Epigenetic regulators could regulate lipid peroxidation by affecting the expression of ferroptosis‐related genes, thereby regulating the level of ferroptosis to impact the occurrence and development of tumors.^58^ As an important chromatin remodeling enzyme, LSH could act as an essential inhibitor of ferroptosis in the tumorigenesis of lung cancer through interacting with WD repeat domain 76, thereby activating the lipid metabolism‐related gene glucose transporter 1, the ferroptosis‐related gene stearoyl‐CoA desaturase 1 and fatty acid desaturases 2, and LSH can be recruited to the promoter region of SLC7A11 to enhance the transcription of SLC7A11, leading to the inhibition of ferroptosis in leukemia.^24^, ^26^

As a unique member of DUBs that could recognize unanchored polyubiquitin specifically, USP5 participates in many biological processes such as DNA repair, development, and stress response.^35^, ^37^, ^59^ And USP5 could regulate the stability of many tumorigenesis‐associated proteins, such as forkhead box M1 (FoxM1), MAF bZIP transcription factor (c‐Maf), Tu translation elongation factor, mitochondrial, and snail family transcriptional repressor 2 (SNAI2) to promote the cancer progression.^42^, ^44^, ^60^, ^61^ However, the function of USP5 in ferroptosis is still unclear. In this study, we showed that USP5 could inhibit the ferroptosis through the stabilization of LSH. Moreover, degrasyn could reduce USP5 protein expression in a dose‐dependent manner. Meantime, the treatment of degrasyn could target USP5 to increase the ubiquitin on LSH, thereby promoting the ferroptosis to suppress the HCC progression. In addition, we reveal that only USP5‐WT, but not USP5‐C335A, could inhibit the ferroptosis by deubiquinating LSH.

In general, we identify USP5 as a DUB of LSH that suppresses the ferroptosis of liver cancer cells to promote cell proliferation and tumor growth through stabilizing LSH protein. Our study reveals a new regulation mechanism of LSH and the effectively suppressive impact of degrasyn on HCC progression, indicating that USP5 might be further developed into a potential therapeutic target in HCC (Figure 7F). However, the specific regulatory mechanism of LSH on SLC7A11 has not been involved in this study. Moreover, whether the inhibitory effect of USP5 on ferroptosis of cancer cells is a common phenomenon in pan‐cancer remains to be further explored.

## 4 MATERIALS AND METHODS

### 4.1 Cell culture, plasmids, sgRNAs, chemicals, and antibodies

The Cancer Research Institute of Central South University provided liver cancer cell lines HepG2, LM3, and Hep3B, normal immortal liver cells L‐02, and human embryonic kidney epithelial cells 293T. In DMEM media (Gibco) with 10% fetal bovine serum (FBS), all cell lines were grown and kept at 37°C with 5% CO_2_. Only cells that had been passed a minimum of 10 times following the first resurrection from cryopreservation and were free of mycoplasma contamination were used.

USP5 cDNA clones were purchased from Vigene Biosciences. By putting USP5 cDNA, USP5 mutant cDNA, or cDNAs encoding these fragments into the pLVX‐EF1‐IRES‐Puro vector (631988; Clontech), the USP5 overexpression plasmid, USP5 mutant plasmid, and truncated Flag‐LSH fragments were created. The MYC‐Ub‐WT, ‐K6, ‐K11, ‐K27, ‐K29, ‐K33, ‐K48, ‐K63, ‐K48R, and ‐K63R were gifts from Professor Pinglong Xu, Life Sciences Institute and Innovation Center for Cell Signaling Network, Zhejiang University, Hangzhou, Zhejiang 310027, China. The sequences of sgRNA targeting USP5 were presented in Table S1. Using Lipofectamine 2000, plasmids were transfected, and 1.5 mg/mL puromycin was used to select colonies that had stable expression.

RSL‐3 (S8155) and ferrostatin‐1 (S7243)^62^ were purchased from Selleck. CHX (Sigma‐Aldrich), MG132 (S2619, Selleck), CQ (C6628, Sigma‐Aldrich), and degrasyn (Topscience) were also used in the cell experiments.

The primary antibodies utilized in this study were purchased commercially: USP5 (10473‐1‐AP, Proteintech), LSH (sc‐46665, Santa Cruz Biotechnology), β‐actin (A5441, Sigma), ACSL4 (sc‐271800, Santa Cruz Biotechnology), SLC7A11 (26864‐1‐AP, Proteintech), GPX4 (A1933, ABclonal), Ubiquitin (10201‐2‐AP, Proteintech), and Flag‐tag (F1804, Sigma).

### 4.2 Cell proliferation, migration, and colony formation assays

Cell viability was measured by CCK8 kit according to its instructions. For the migration assays, 4 × 10^4^ cells with different treatments were seeded in chamber. The cells on the chamber's upper surface were removed 24 h later, and the chamber was then preserved with methanol and stained with 0.5% crystal violet. Approximately 1000 cells were planted in each well of six‐well plates for the colony formation assays. Cells were methanol fixed and stained with 0.5% crystal violet after about 2 weeks.

### 4.3 Western blot analysis and coimmunoprecipitation (Co‐IP) assay

In IP lysis buffer augmented with a protease inhibitor cocktail, the collected cells were lysed. Total protein from cell lysis was transferred to a PVDF membrane after being separated by an SDS–polyacrylamide gel. Primary antibodies were used to incubate membrane at 4°C overnight. Bands were visualized using the ChemiDoc XRS+ image‐forming system.

For immunoprecipitation, cells were collected, lysed in IP buffer with a cocktail of protease inhibitors. Complete cell lysis was precleared with 10 μL of Dynabeads Protein G (10004D, Invitrogen), and subsequently immunoprecipitated with corresponding antibodies overnight at 4°C on a rotator. The lysates underwent an additional 2 h of incubation at 4°C with the addition of beads. Following three cold IP buffer washes, the immunocomplexes were separated using SDS–PAGE and immunoblotted using the indicated antibodies.

### 4.4 RT‐qPCR

TRIzol (Takara) was used to isolate the total RNA, and a PrimeScript RT reagent kit (Takara), including gDNA Eraser, was used to produce the cDNA. Utilizing the FastStart Universal SYBR Green Master and the ABI 7500, RT‐qPCR was carried out. β‐Actin was used to standardize relative gene expression levels. Table S2 includes a list of the primer sequences for RT‐qPCR.

### 4.5 Histology and immunohistochemistry

Antigen retrieval was finished by heating tissues in sodium citrate following normal deparaffinization with xylene and hydration with ethanol of decreasing concentrations. Following that, tissues’ endogenous peroxidase was blocked by incubation with 3% hydrogen peroxide. The tissues were then treated with the appropriate secondary antibody at room temperature (RT) for 30 min after being exposed to the primary antibodies, which were anti‐USP5 (1:200) and anti‐LSH (1:200) overnight at 4°C. A pathologist verified the liver cancer biopsies submitted by the Department of Pathology at Xiangya Hospital (Dr. Desheng Xiao of Xiangya Hospital).

### 4.6 Deubiquitination assay

In 293T, HepG2, LM3, and Hep3B cells, a deubiquitination assay was conducted. HA‐Ub, LSH, Flag‐USP5, or USP5‐C335A plasmid were transiently transfected into 293T cells. Two days after transfection, the cells were exposed to 20 μM MG132 (Selleck, S2619) for 12 h before being harvested. The cells were then immunoprecipitated to separate HA‐ubiquitinated LSH. The level of LSH ubiquitination was detected using western blot. The abovementioned experimental procedures were carried out in cells stable overexpressing USP5 (HepG2, LM3) or knocking out USP5 (Hep3B).

### 4.7 Measurement of lipid ROS and total GSH

Cells were treated for 24 h with 6 μM RSL‐3 for the lipid ROS assay, after which they were trypsinized and resuspended in DMEM media with 10% FBS and 10 μM of C11‐BODIPY (D3861, Thermo Fisher). The samples were then incubated at 37°C and 5% CO_2_ for 30 min while being shielded from light. To get rid of extra C11‐BODIPY, PBS was used to wash the cells twice. Dr. Shuang Liu oversaw the use of a flow cytometer (Fortessa, BD Biosciences) with fluorescein isothiocyanate green channel and Texas red channel to measure the fluorescence of C11‐BODIPY581 = 591.

For the detection of Total GSH, after 24 h of incubation with 6 μM RSL‐3, cells were digested and collected in centrifuge tubes, and three volumes of ice‐cold 5% metaphosphoric acid were added immediately then frozen and thawed twice using liquid nitrogen and 37°C water. The supernatant was utilized for total GSH measurement in accordance with the protocol after 10 min of centrifugation at 12,000 rpm and 4°C.

### 4.8 Cell imaging of ferroptosis

In 6‐well plates, 5 × 10^5^ cells were planted each well. Images were captured using a microscope after 24 h of incubation with 6 μM RSL‐3.

### 4.9 Immunofluorescence

On a 24‐well plate with glass coverslips, cells were sown. The cells were frozen in methanol for 10 min at −2.0°C after 24 h of incubation and then blocked in PBS with donkey serum (S9100, Solarbio). Cells were then incubated with primary antibodies against USP5 (10473‐1‐AP, Proteintech) and LSH (sc‐46665, Santa Cruz Biotechnology) overnight at 4°C, followed by an hour‐long incubation at RT with Alexa Fluor 488 donkey anti‐rabbit and Alexa Fluor 568 donkey anti‐mouse (Life Technologies), both of which were light‐protected. The nuclei were stained with DAPI (2273641, Invitrogen). A Leica fluorescence microscope was used to take the fluorescence pictures.

### 4.10 Nude mice and study approval

The Hunan SJA Laboratory Animal Co., Ltd. supplied the female nude mice at the age of 4 weeks. The Xiangya School of Medicine of Central South University's Institutional Animal Care and Use Committee approved the use of animals in experiments, and the experiments were carried out in accordance with the law and federal regulations for the care and protection of animals (Permit No. 2020sydw0117). 3 × 10^6^ LM3/Hep3B/HepG2 cells were injected subcutaneously into the back of each mouse. As the trial progressed, tumor sizes were assessed every 3 days until the 25th day after injection for LM3 and Hep3B cells and every 2 days until the 20th day after the injection for HepG2 cells. Weighting, protein and RNA extraction, or 10% formalin fixation and paraffin embedding of tumors were all done.

### 4.11 Statistical analyses

All tests were carried out at least three times, with the exception of those using naked mice. Data were presented as the mean ± SD or SEM, utilizing GraphPad Prism 8.0 and Microsoft Excel 2019 for all statistical analysis. To compare two or more groups, Student's *t* test and the analysis of variance were used, respectively. Statistical significance was defined as a *p*‐value <0.05.

## AUTHOR CONTRIBUTIONS

Yongguang Tao and Desheng Xiao designed and supervised this research. Bokang Yan, Jiaxing Guo, Zuli Wang, Shuang Liu, and Jieling Ning performed the experiments. Desheng Xiao and Kuan Hu were in charge of collecting the clinical samples. Ling Chen and Ying Shi provided helpful discussions. Bokang Yan, Zuli Wang, Long Shu, and Desheng Xiao analyzed and interpreted the data. Bokang Yan wrote this paper with the help of Haiyan Wang, Lingqiang Zhang, Yongguang Tao, and Desheng Xiao. This manuscript was approved by all authors.

## CONFLICT OF INTEREST STATEMENT

The authors declare no conflicts of interest.

## ETHICS STATEMENT

Animal experiments were performed with the approval of the Institutional Animal Care and Use Committee of the Xiangya School of Medicine of the Central South University (Permit No. 2020sydw0117) and in compliance with the legal mandates and federal guidelines for animal protection and maintenance. All patients who donated tissues for IHC were provided with the informed consent.

## Supporting information

Supporting Information

## Data Availability

Correspondence and requests for materials or data should be addressed to Yongguang, Tao or Desheng, Xiao.
