# Supplementary material for: The ubiquitin‐specific protease 5 mediated deubiquitination of LSH links metabolic regulation of ferroptosis to hepatocellular carcinoma progression
Source: MedComm (2020). 2023 Jul 22;4(4):e337. doi: 10.1002/mco2.337 (PMC10363799; doi:10.1002/mco2.337)
Supplement: Supplementary file 1 — Supporting Information [file MCO2-4-e337-s001.docx]

Supporting Information for

**The USP5-mediated deubiquitination of LSH links metabolic regulation of ferroptosis to hepatocellular carcinoma progression**

Bokang Yan1,2,3, Jiaxing Guo1,3, Zuli Wang1,3, Jieling Ning3, Haiyan Wang1,3, Long Shu3, Kuan Hu3,4, Ling Chen1,3, Ying Shi1,3, Lingqiang Zhang5, Shuang Liu6, Yongguang Tao1,3,7*, Desheng Xiao1,8*

*Correspondence: Desheng Xiao ([xdsh96@csu.edu.cn)](Supplementary-USP5 LSH.docx) or Yongguang Tao ([taoyong@csu.edu.cn](mailto:taoyong@csu.edu.cn))

**This file includes:**

Figures. S1 to S6

Tables S1 to S2

**
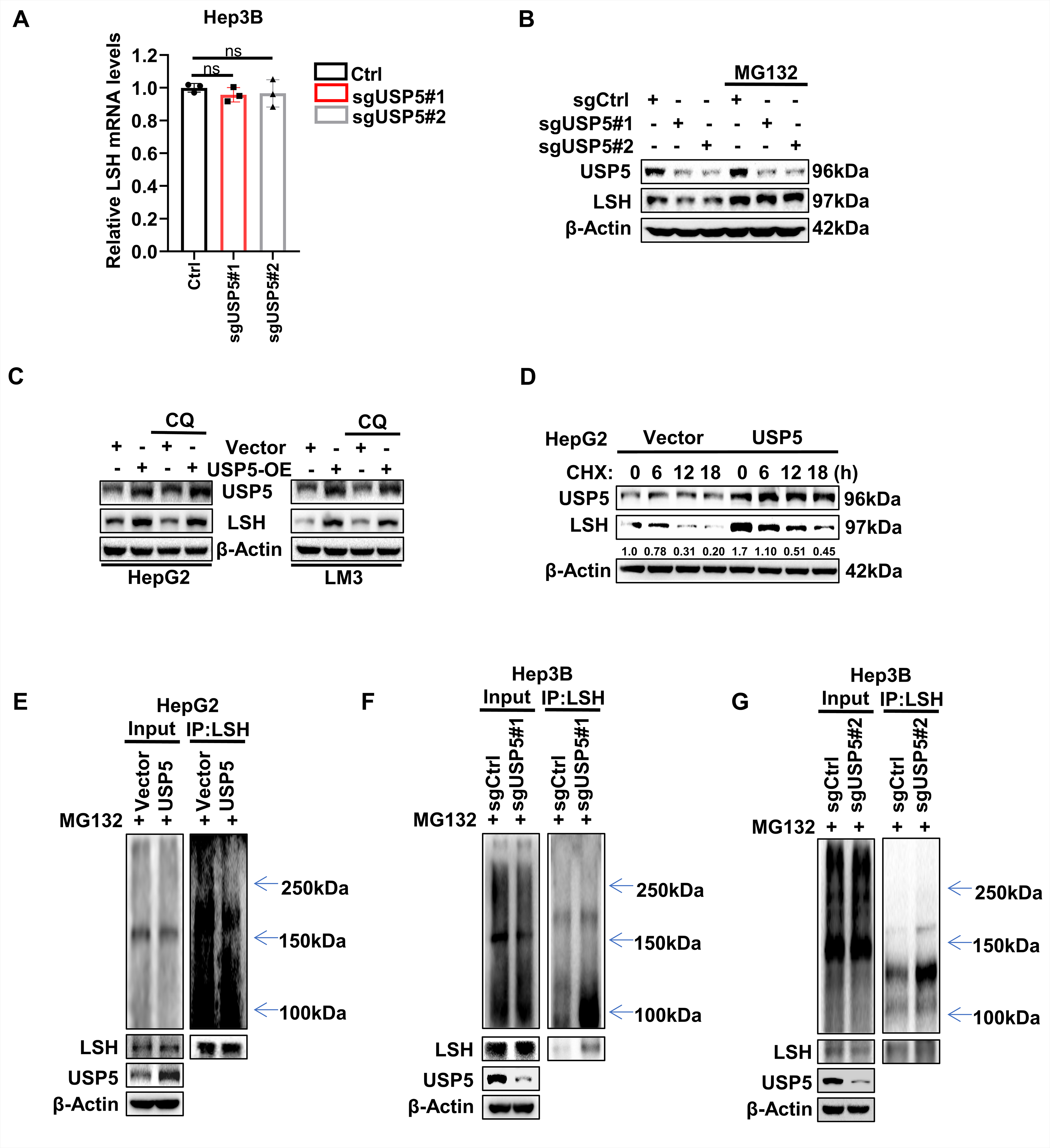
Figure S1. USP5 stabilizes the LSH protein through deubiquitination.** (A) qRT-PCR analysis of LSH in Hep3B cells knockout of USP5. (B) Hep3B cells knockout of USP5 were treated with or without MG132 (20μM, 12h), then western blot was used to test the protein level of LSH. (C) HepG2 and LM3 cells overexpressing USP5 were treated with or without chloroquine (CQ) (10μM, 24h), then western blot was used to test the protein level of LSH. (D) HepG2 cells stably overexpressing USP5 were treated with cycloheximide (CHX, 10 mg/ml) for the indicated time followed by WB. (E-G) HepG2 cells stably overexpressing USP5 or Hep3B cells knockout of USP5 were treated with MG132 (20μM, 12h) before collection. LSH was immunoprecipitated with anti-LSH and immunoblotted with anti-Ub. ns nonsignificant (p > 0.05).

**
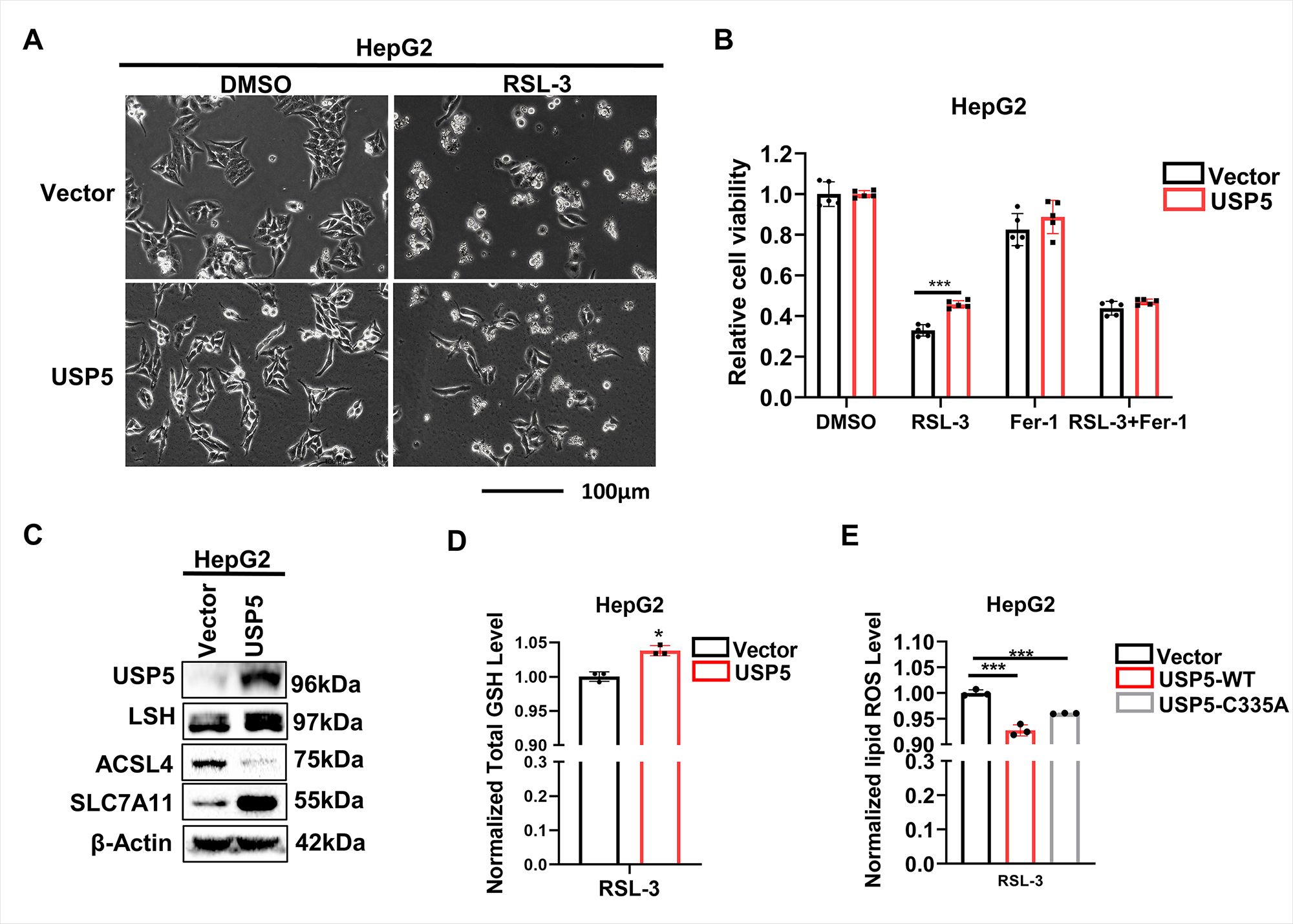
Fig S2. Overexpression of USP5 inhibits the ferroptosis level of HepG2 cells.** (A) Representative phase-contrast images of HepG2 cells stably overexpressing USP5 treated with RSL-3 (10μM, 24h). Scale bars, 100 μm. (B) CCK8 assays were used to analyze the responses of HepG2 cells overexpressing USP5 to ferrostatin (10μM, 24h) and RSL-3 (10μM, 24h). (C) Western blot for detecting the expression level of LSH and ferroptosis-related proteins in HepG2 cells stably overexpressing USP5. (D) The levels of total GSH in HepG2 cells stably overexpressing USP5 were analyzed. (E) The levels of lipid ROS in HepG2 cells transfected with USP5-WT or USP5-C335A were analyzed. * P<0.05, *** P<0.001.

**
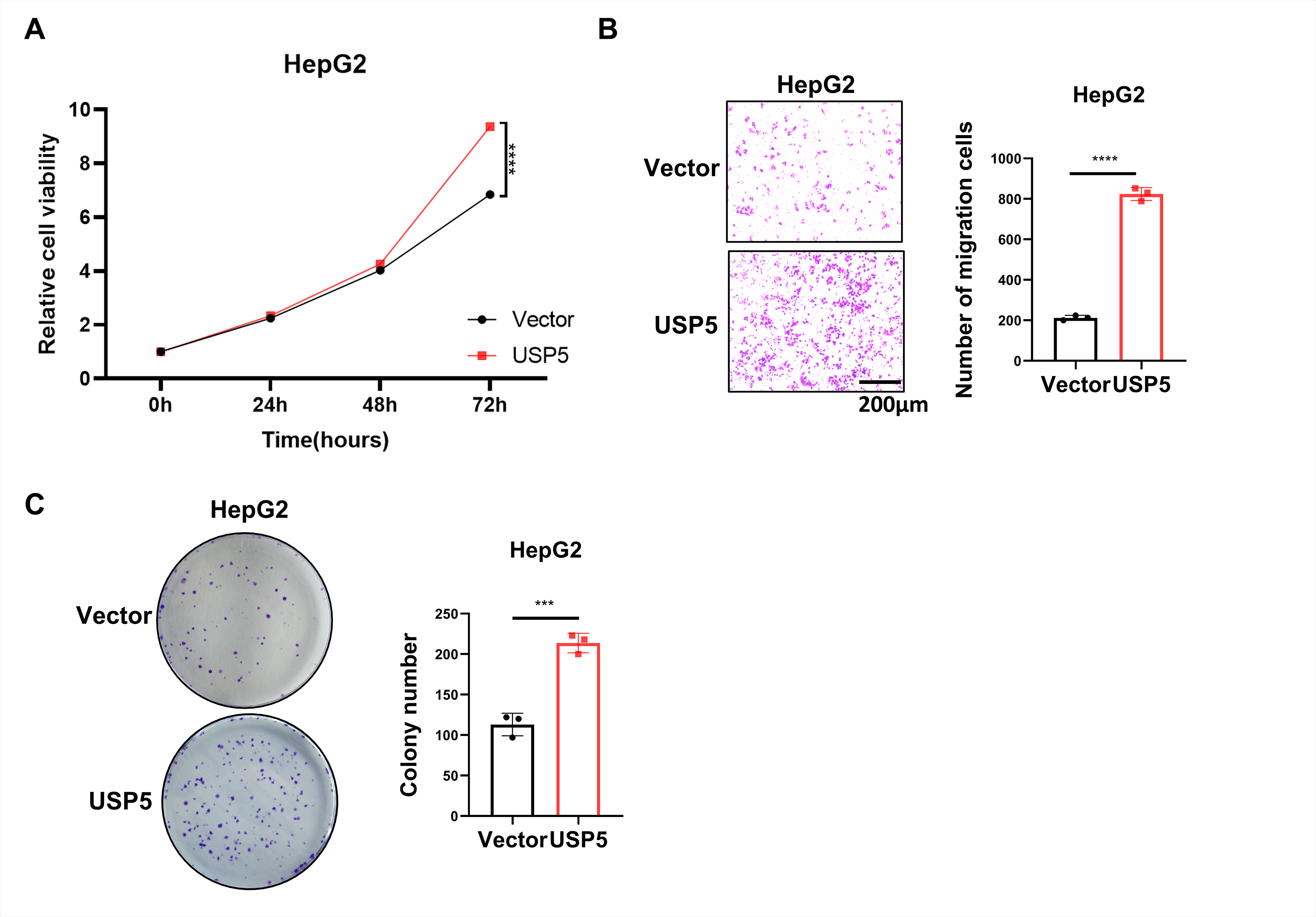
Fig S3. Overexpression of USP5 promotes the cell proliferation, cell migration and colony formation ability of HepG2 cells.** (A-C) The CCK8 assay (A), transwell assay (B), and colony formation assay (C) of HepG2 cells stably overexpressing USP5. Scale bars, 200 μm. *** P<0.001, **** P<0.0001.

**
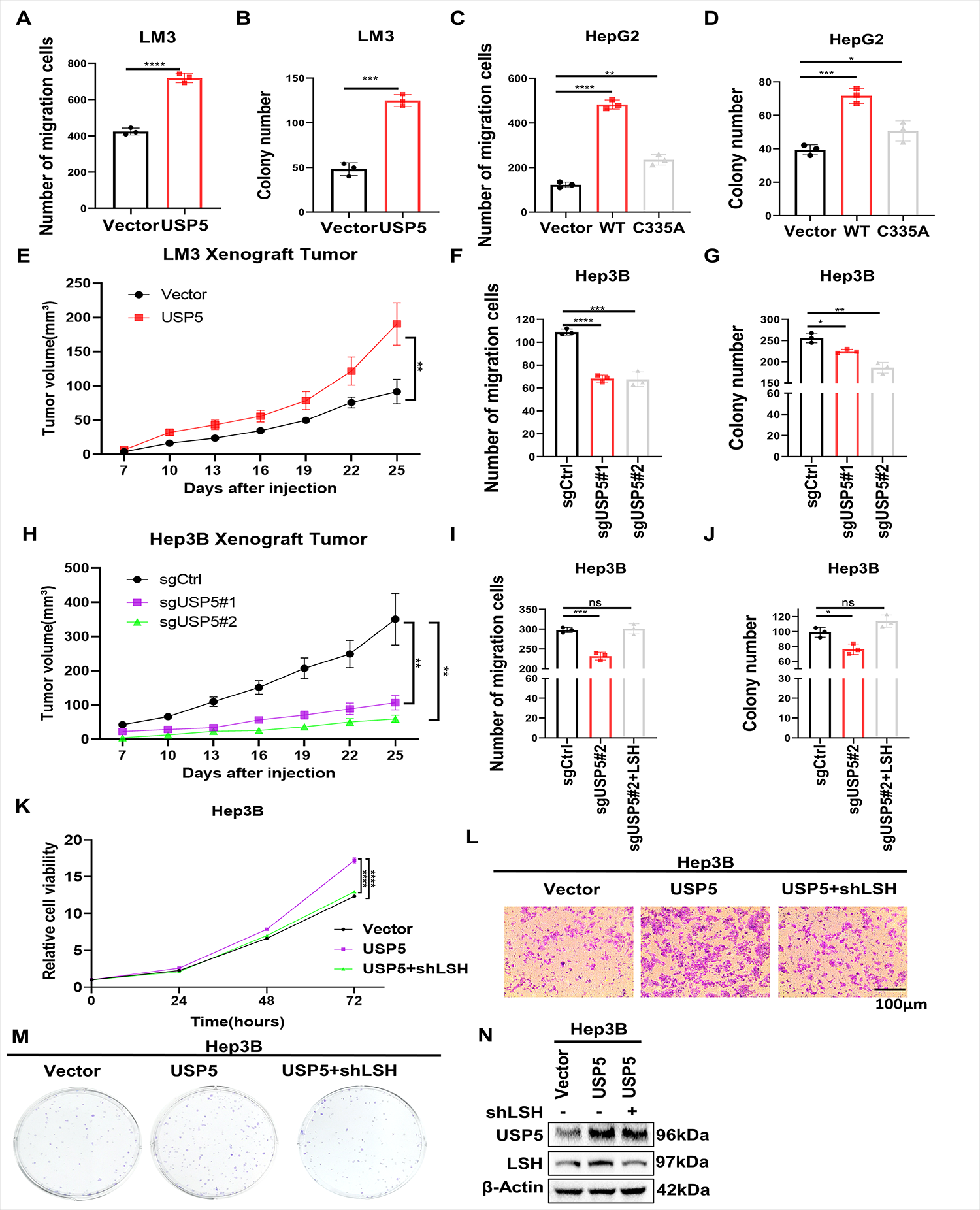
Fig. S4 USP5 promotes tumor growth partially through stabilizing LSH.** (A, B) The transwell assay (A) and colony formation assay (B) of LM3 cells stably overexpressing USP5. (C, D) The transwell assay (C) and colony formation assay (D) of HepG2 cells transfected with USP5-C335A. (E) LM3 cells stably overexpressing USP5 were transplanted on nude miced, and tumor formation was monitored at the indicated times (n = 6 mice per group). (F, G) The transwell assay (F) and colony formation assay (G) of Hep3B cells knockout of USP5. (H) Hep3B cells knockout of USP5 were transplanted on nude miced, and tumor formation was monitored at the indicated times (n = 6 mice per group). (I, J) The transwell assay (I) and colony formation assay (J) of Hep3B cells knockout of USP5 with LSH overexpression. (K-M) The CCK8 assay (K), transwell assay (L) and colony formation assay (M) of Hep3B cells overexpressing USP5 with LSH knockdown. Scale bars, 100 μm. (N) Western blot was used to detect the expression level of LSH and USP5 in Hep3B cells overexpressing USP5 with LSH knockdown. ns nonsignificant (p > 0.05), * P<0.05, ** P<0.01, *** P<0.001, **** P<0.0001.

**
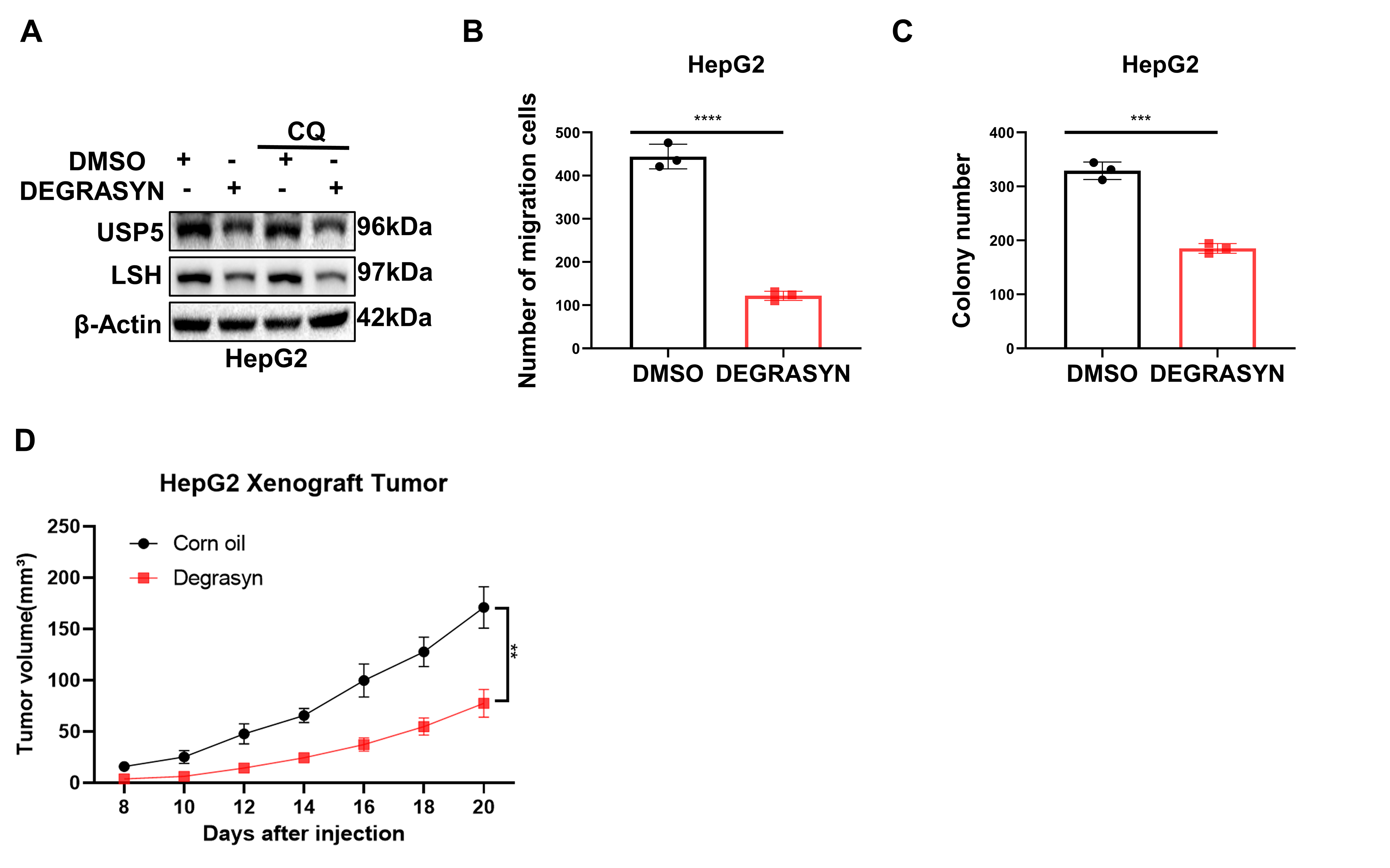
Fig. S5 Degrasyn promotes ferroptosis to inhibit tumor progression via targeting of USP5.** (A) HepG2 cells treated with degrasyn were treated with or without CQ (10μM, 24h), then western blot was used to test the protein level of LSH. (B, C) The transwell assay (B), and colony formation assay (C) of HepG2 cells treated with DMSO or degrasyn. (D) The parental HepG2 cells were transplanted on nude mice treated with corn oil or degrasyn intraperitoneally (25mg/kg, 3 times/week), and tumor formation was monitored at the indicated times (n = 6 mice per group). ** P<0.01, *** P<0.001, **** P<0.0001.

**
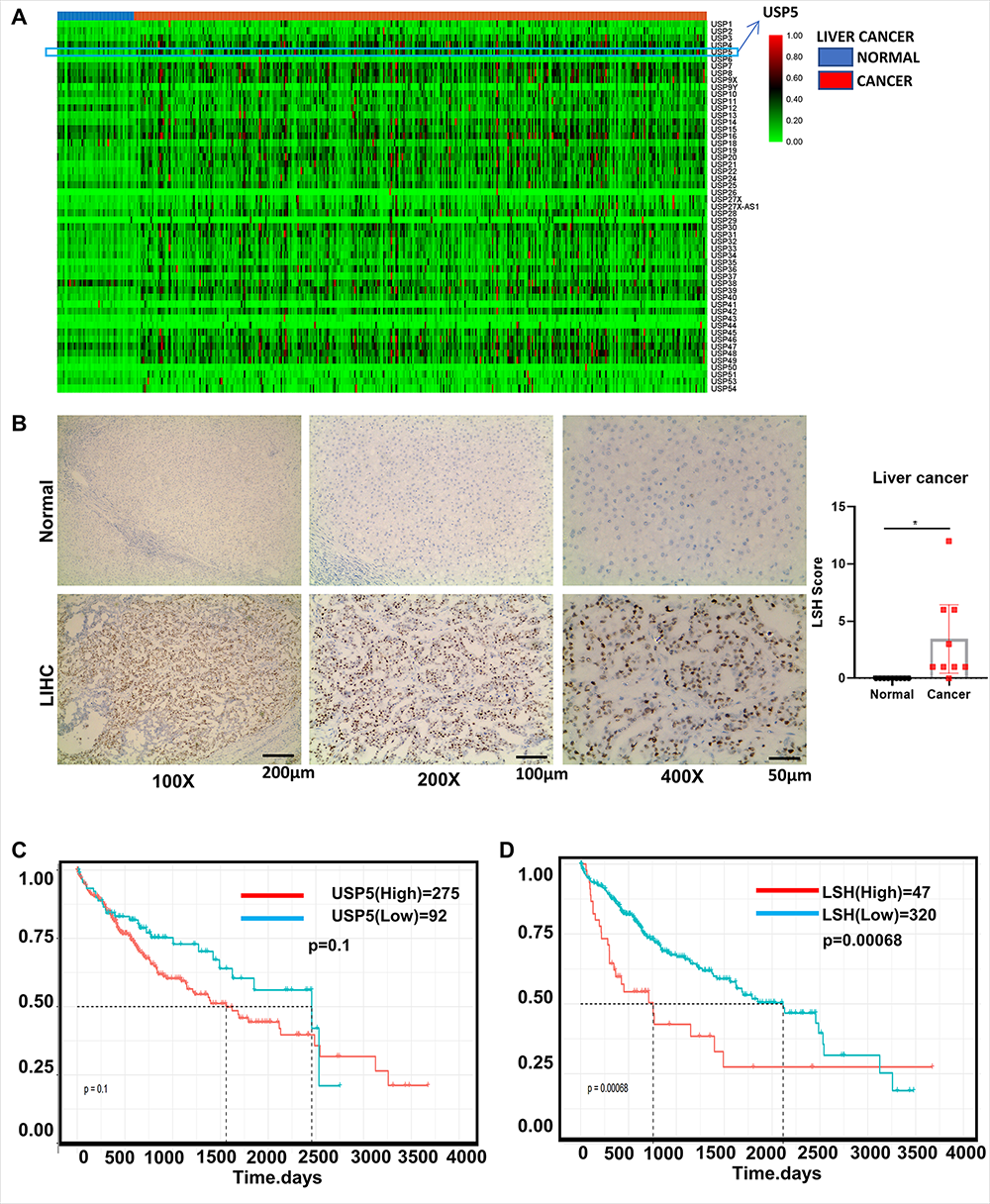
Fig S6. USP5 and LSH are upregulated and associated with poor survival in HCC.** (A) Heatmap of the mRNA expression of USPs family in TCGA HCC samples (n=374) and normal liver tissues (n=50). (B) IHC test of LSH protein expression in 10 pairs of clinical samples (magnification, ×100 scale bar = 200 μm; magnification, ×200 scale bar = 100 μm; magnification, ×400 scale bar = 50 μm). (C) Kaplan–Meier curve showing the overall survival rate of TCGA LIHC samples classified by USP5 expression (n=367). (D) Kaplan–Meier curve showing the overall survival rate of TCGA LIHC samples classified by LSH expression (n=367).

**Table. S1** sgRNA sequence used in this paper

| Name | Source | Target Sequences |
| --- | --- | --- |
| USP5-sg#1 | ORIGENE | F:CACCGAGCGAGTCTACTTGCACCTC  R:AAACGAGGTGCAAGTAGACTCGCTC |
| USP5-sg#2 | ORIGENE | F:CACCGGTGCAAGTAGACTCGCTGGC  R:AAACGCCAGCGAGTCTACTTGCACC |

**Table. S2** Sequences for RT-PCR primers

| Name | Gene ID | Primer |
| --- | --- | --- |
| USP5 | 8078 | F:GAAGTGTTCCGCTTCTTGGTGG  R:TTGCCGCTTCTTCTCCTCGTAC |
| LSH | 3070 | F:GATTTTGGATCGAATGCTGCCAG  R:ATGGACCCATCAAGCCTGCTGA |
